# Therapeutic Effects of Naringin in Rheumatoid Arthritis: Network Pharmacology and Experimental Validation

**DOI:** 10.3389/fphar.2021.672054

**Published:** 2021-05-14

**Authors:** Yirixiati Aihaiti, Yong Song Cai, Xiadiye Tuerhong, Yan Ni Yang, Yao Ma, Hai Shi Zheng, Ke Xu, Peng Xu

**Affiliations:** ^1^ Department of Joint Surgery, Xi’an Jiaotong University Affiliated HongHui Hospital, Xi’an, China; ^2^ Department of Thoracic Surgery, Xi’an Jiaotong University Affiliated HongHui Hospital, Xi’an, China; ^3^ Department of Rehabilitation, Xi’an Jiaotong University Affiliated HongHui Hospital, Xi’an, China

**Keywords:** rheumatoid arthritis, naringin, network pharmacology, PI3k/Akt signaling pathway, MAPK/ERK signaling pathway

## Abstract

Rheumatoid arthritis is a chronic autoimmune disease characterized by persistent hyperplasia of the synovial membrane and progressive erosion of articular cartilage. Disequilibrium between the proliferation and death of RA fibroblast-like synoviocytes (RA-FLSs) is the critical factor in progression of RA. Naringin has been reported to exert anti-inflammatory and antioxidant effect in acute and chronic animal models of RA. However, the therapeutic effect and underlying mechanisms of naringin in human RA-FLS remain unclear. Based on network pharmacology, the corresponding targets of naringin were identified using SwissTargetPrediction database, STITCH database, and Comparative Toxicogenomics Database. Deferentially expressed genes (DEGs) in RA were obtained from the GEO database. The protein–protein interaction (PPI) networks of intersected targets were constructed using the STRING database and visualized using Cytoscape. Gene Ontology (GO) and Kyoto Encyclopedia of Genes and Genomes (KEGG) pathway enrichment analyses were performed, and the pathways directly related to pathogenesis of RA were integrated manually. Further, *in vitro* studies were carried out based on network pharmacology. 99 target genes were intersected between targets of naringin and DEGs. The PPI network and topological analysis indicated that IL-6, MAPK8, MMP-9, TNF, and MAPK1 shared the highest centrality among all. GO analysis and KEGG analysis indicated that target genes were mostly enriched in (hsa05200) pathways in cancer, (hsa05161) hepatitis B, (hsa04380) osteoclast differentiation, (hsa04151) PI3K-Akt signaling pathway, and (hsa05142) Chagas disease (American trypanosomiasis). *In vitro* studies revealed that naringin exposure was found to promote apoptosis of RA-FLS, increased the activation of caspase-3, and increased the ratio of Bax/Bcl-2 in a dose-dependent manner. Furthermore, treatment of naringin attenuated the production of inflammatory cytokines and matrix metalloproteinases (MMPs) in TNF-ɑ–induced RA-FLS. Moreover, treatment of naringin inhibited the phosphorylation of Akt and ERK in RA-FLS. Network pharmacology provides a predicative strategy to investigate the therapeutic effects and mechanisms of herbs and compounds. Naringin inhibits inflammation and MMPs production and promotes apoptosis in RA-FLS *via* PI3K/Akt and MAPK/ERK signaling pathways.

## Introduction

Rheumatoid arthritis (RA) is an autoimmune disease characterized with chronic synovitis along with progressive cartilage and bone erosion. Persistent inflammation eventually leads to synovial hyperplasia, joint deformity, and disability (Firestein and Mcinnes, 2017). The average prevalence of RA is estimated to range within 0.5–1.0% globally (Gottenberg et al., 2019). Serological parameters, such as anti-citrullinated protein antibodies (ACPAs) and rheumatoid factor (RF), are the distinctive features of RA, and immunological abnormalities precede the development of RA (Veale et al., 2017). Genetic factors, infection, and environmental factors have been reported to partly contribute to this, but the actual etiology of RA remains unclear (Scott et al., 2010). The present treatment of RA aims to control the inflammatory reaction and relieve joint pain. Disease-modifying antirheumatic drugs (DMARDs) have the tendency for the inhibition of inflammatory and damaging events in RA patients (Scott et al., 2010). In recent years, biological agents that target inflammatory cytokines, such as IL-1β, IL-6, and TNF-α, have been widely used, and these have substantially improved the RA therapy.

Naringin (4′,5,7-trihydroxyflavanone-7-neohesperidoside) is a flavanone glycoside formed from the flavanone naringenin. Naringin is one of the main active components of citrus fruits (Chen et al., 2016). Naringin has been widely used for the treatment of various cancers, osteoporosis, bone regeneration, and diabetes. Naringin exerts osteogenic activity by promoting osteoblastic functions and suppressing osteoclastogenesis (Ang et al., 2011). Furthermore, naringin has been reported to induce apoptosis and inhibit proliferation in various tumor cells in a dose-dependent manner, including human cervical cancer (SiHa) cells and bladder cancer cells (Kim et al., 2008; Ramesh and Alshatwi, 2013). In addition, naringin has attracted wide attention in the treatment of chronic inflammatory diseases. Naringin becomes a novel approach for treatment of arthritis. *In vivo* and *in vitro* experiments revealed that naringin attenuated stiffness and joint pain, reduced the secretion of pro-inflammatory cytokines, and prevented subchondral bone sclerosis in the monosodium iodoacetate (MIA)–induced OA model and anterior cruciate ligament transection (ACLT)–induced OA model in mice (Zhao et al., 2016; Xu et al., 2017).

**FIGURE 1:**
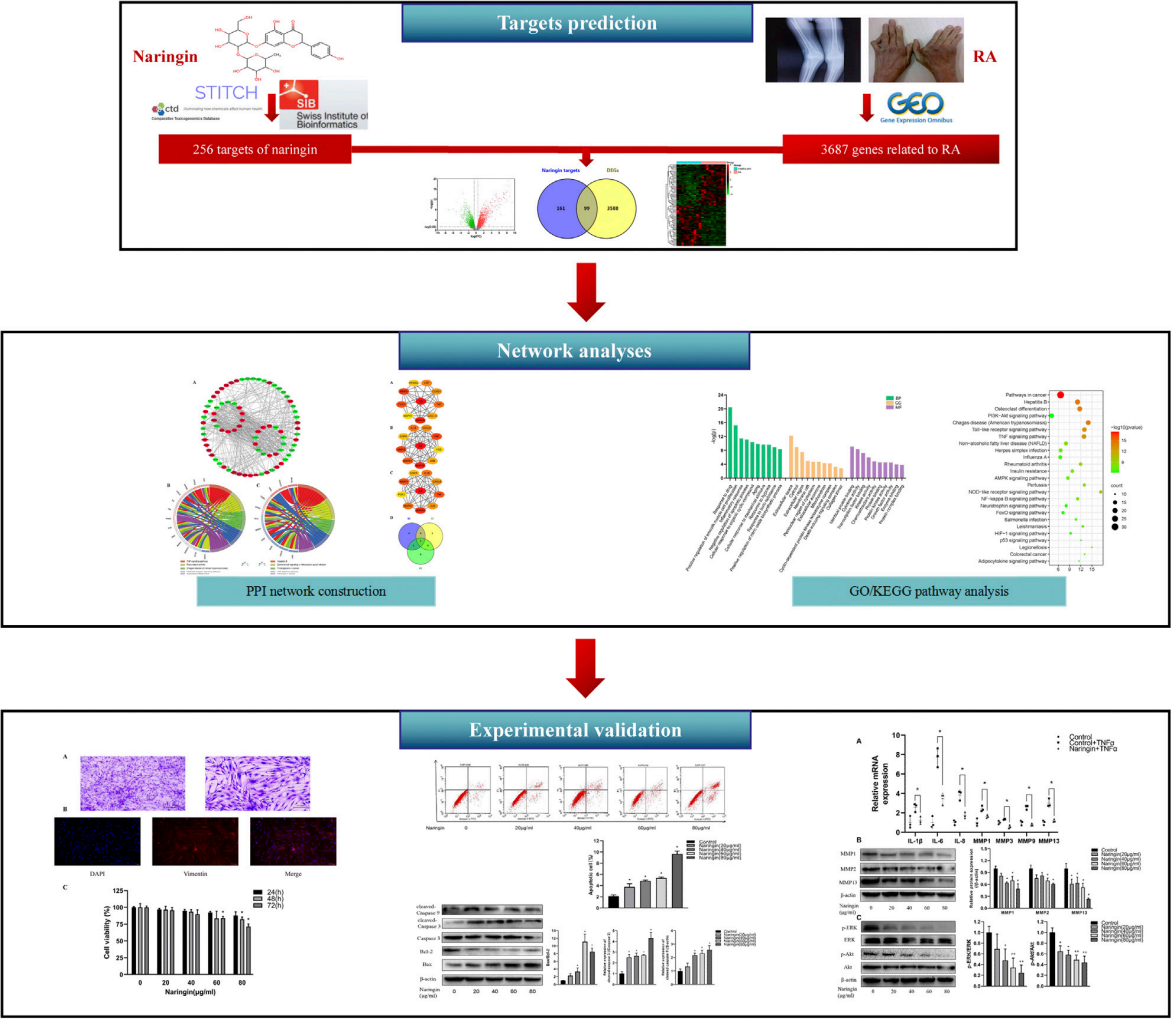
Technical strategy of the current study.

Network pharmacology is a novel strategy for new drug exploration based on the therapeutic targets of herbs and compounds, which simultaneously embraces efforts to improve clinical efficacy and understands the side effects and toxicity. The network pharmacology method is considered as the next paradigm of drug development (Hopkins, 2008), which updates the existing research paradigm of “drug-single target” into a new paradigm of “drug–disease multiple target gene-signaling pathway” (Huang et al., 2014). Therefore, in the present research, network pharmacology was used to excavate the therapeutic targets and related pathways of naringin against RA. The main purposes of present study were 1) to screen the potential targets of naringin and DEGs in RA synovial tissue; 2) to analyze the underlying mechanisms of naringin against RA using network pharmacology; 3) to verify anti-inflammatory and proapoptotic effects and underlying pathway of naringin in RA-FLS. The present study may provide a new therapeutic approach for the treatment of RA. The technical strategy of the current study is shown in Figure 1.

## Materials and Methods

### Chemical Reagents

Naringin was purchased from Sigma, dissolved in DMSO, and diluted in the DMEM. Fetal bovine serum (FBS) and antibiotics (streptomycin/penicillin) were obtained from Gibco. Dulbecco’s Modified Eagle Medium (DMEM) and trypsin were purchased from Hyclone and dimethyl sulfoxide (DMSO) from Beijing Suolaibao Technology Co., LTD. (Beijing, China). Primary antibodies caspase-3 and cleaved caspase-3 were purchased from Abcam. Bcl-2, Bax, Akt, *p*-Akt, ERK, *p*-ERK, ACTB, and anti-rabbit IgG horseradish conjugate secondary antibody were purchased from Cell Signaling. MMP-1, MMP-13, MMP-2, and vimentin were procured from Proteintech Group, Inc., (Wuhan, China). Nitrocellulose membranes and ECL detection reagents were purchased from Millipore (Millipore, Bredford, PA, United States). Stripping buffer for reusing NC-membrane was purchased from Beyotime (Beyotime Biotech, Nanjing, China). Human recombinant tumor necrosis factor-α (hrTNF-α) was purchased from Sigma. Primers used in qPCR were designed and synthesized by Sangon Biotech (Table 1). TRIzol reagent was purchased from Invitrogen (Invitrogen, Carlsbad, CA, United States). RevertAid First Strand cDNA Synthesis Kit was obtained from Thermo Scientific (K1621, Thermo Scientific, Waltham, MA, United States). ChamQ Universal SYBR qPCR Master Mix was purchased from Vazyme (Vazyme Biotech, Nanjing, China). CCK-8 kit and RIPA lysis buffer were purchased from Beyotime (Beyotime Biotech, Nanjing, China).

**TABLE 1:** Primer sequences for real-time PCR.

Gene name	Primer sequences	
ACTB	Forward	GGC​CAA​CCG​CGA​GAA​GAT​GAC
	Reverse	GGA​TAG​CAC​AGC​CTG​GAT​AGC​AAC
IL-1	Forward	GCC​AGT​GAA​ATG​ATG​GCT​TAT​T
	Reverse	AGG​AGC​ACT​TCA​TCT​GTT​TAG​G
IL-6	Forward	CAC​TGG​TCT​TTT​GGA​GTT​TGA​G
	Reverse	GGA​CTT​TTG​TAC​TCA​TCT​GCA​C
IL-8	Forward	AAC​TGA​GAG​TGA​TTG​AGA​GTG​G
	Reverse	ATG​AAT​TCT​CAG​CCC​TCT​TCA​A
MMP-1	Forward	AGA​TTC​TAC​ATG​CGC​ACA​AAT​C
	Reverse	CCT​TTG​AAA​AAC​CGG​ACT​TCA​T
MMP-3	Forward	TGA​GGA​CAC​CAG​CAT​GAA​CC
	Reverse	ACT​TCG​GGA​TGC​CAG​GAA​AG
MMP-9	Forward	CAG​TAC​CGA​GAG​AAA​GCC​TAT​T
	Reverse	CAG​GAT​GTC​ATA​GGT​CAC​GTA​G
MMP-13	Forward	CAC​TTT​ATG​CTT​CCT​GAT​GAC​G
	Reverse	TCT​GGC​GTT​TTT​GGA​TGT​TTA​G

### Naringin Target Prediction and RA-Related Target Screening

The chemical structure and SMILES (simplified molecular input line entry specification) of naringin was obtained from PubChem website (https://pubchem.ncbi.nlm.nih.gov/compound/442428) (Kim et al., 2021). Target prediction of naringin was carried out using the SwissTargetPrediction database (http://www.swisstargetprediction.ch/) (Gfeller et al., 2014), Comparative Toxicogenomics Database (http://ctdbase.org/) (Davis et al., 2021), and STITCH database (http://stitch.embl.de/) (Szklarczyk et al., 2016), while the species was limited to “*Homo sapiens*.” The row gene expression data (GSE55235) of synovial tissue in RA and healthy donors were obtained from the Gene Expression Omnibus dataset in the National Center for Biotechnology Information (https://www.ncbi.nlm.nih.gov/geo/) (Woetzel et al., 2014). The GSE55235 dataset comprised the gene expression data of 10 synovial tissue samples from healthy donors, 10 synovial tissue samples from patients with RA, and 10 synovial tissue samples for patients with OA. “Limma” R package was used to identify DEGs between RA synovial tissue and healthy donors. The cutoff criteria in this analysis was set as *p* < 0.05 and |log FC| >0.5. The intersection of aforementioned targets and DEGs was visualized using the Venny online tool (Sun et al., 2019).

### GO and KEGG Pathway Enrichment Analyses

Intersected target genes from naringin and RA were uploaded to the Database for Annotation, Visualization, and Integrated Discovery (DAVID 6.8; http://david.abcc.ncifcrf.gov/) (Huang et al., 2009) to determine functional term enrichment. Gene Ontology (GO) enrichment analysis includes Biological Process (BP), Molecular Function (MF), and Cellular Component analysis. Kyoto Encyclopedia of Genes and Genomes (KEGG) is a bioinformatics resource for mining significantly altered metabolic pathways enriched in the gene list. GO enrichment analysis (*p* < 0.05) and KEGG pathway analysis (*p* < 0.05) were visualized using the bioinformatics platform (http://www.bioinformatics.com.cn/).

### Protein–Protein Interaction Network Analysis

The PPI network of target genes was obtained from the Search Tool for the Retrieval of Interacting Genes/Proteins (STRING 11.0; https://string-db.org/) (Szklarczyk et al., 2019) database, with minimum required interaction score ≥0.7. The PPI network was visualized using Cytoscape v3.7.2 (Shannon, 2003). In the PPI network, nodes represent the target proteins, while edges represent the predicted or validated interaction between proteins. The molecular complex detection (MCODE) plug-in of Cytoscape was applied to detect subnetworks in the PPI network (degree cutoff ≥2, node score cutoff ≥0.2, K-core ≥ 4, and max depth = 100). Subsequently, KEGG pathway analysis of DEGs in modules was performed using the DAVID database. Topological analysis of target genes was performed using the NetworkAnalyzer plug-in and CytoNCA plug-in of Cytoscape. Target proteins were filtered separately according to the betweenness centrality (BC), closeness centrality (CC), and degree centrality (DC), which were calculated using CytoNCA plug-in. Top 10 genes of each subnetwork were retrieved, and overlapped genes were selected as key targets in the present research.

### Isolation and Identification of Synovial Cells

Synovial biopsies were obtained from RA patients who underwent total knee replacement surgery in Xi’an Jiaotong University affiliated Honghui hospital. All patients fulfilled the 2010 American College of Rheumatology (ACR) diagnostic criteria, and disease active score (DAS)≥3.0 (van der Linden et al., 2011). All the spices and clinical information in this research were collected after providing written informed consents to all patients. The present study was approved by the Clinical Research Ethics Committee of Xi’an Jiaotong University affiliated Honghui Hospital (No.202103062).Fresh synovial tissues were washed with phosphate buffer solution (PBS) for three times and minced into small pieces with ophthalmic scissors. Then spices were digested with 2 mg/ml type I collagenase for 30 min and 0.25% trypsin for 2 h in 37°C. Subsequently, the obtained cell was suspended in DMEM supplemented with 10% fetal bovine serum (FBS), 100 U/ml penicillin, and 100 μg/ml streptomycin at 37°C with 5% CO2. RA-FLSs at passage 3–7 were used for experiments. RA-FLS was fixed with 4% paraformaldehyde and stained with 5% crystal violet for morphological observation, with the vimentin antibody for immunofluorescence staining, and were observed under a fluorescence microscope.

### Cell Viability Assay

Cell viability was measured using CCK-8 assay. RA-FLS was seeded into the 96-well plate in the concentration of 3 × 10^3^ cell per well. After incubating in a cell incubator overnight, RA-FLS was exposed to naringin in concentrations of 0–80 μM for 24–72 h according to previous research of naringin (Yang et al., 2020a). 10 μl of CCK-8 solution was added to each well of the plate. The plate was incubated for 4 h in the incubator. The optical density (OD) was measured at 450 nm wavelength with a microplate reader. Each experiment was repeated three times. Cell viability was calculated using formula: $\left({\text{OD}}_{\text{Experimental group}}{-\text{OD}}_{\text{Blank}}\right)/\left({\text{OD}}_{\text{Control group}}{-\text{OD}}_{\text{Blank}}\right)$.

### Total RNA Extraction and Real-Time PCR

RA-FLS was treated with different concentrations of naringin; after overnight incubation, RA-FLS was treated with 10 ng/ml tumor necrosis factor alpha (TNF-α) to establish an inflammatory model of RA. Total RNA of RA-FLSs was extracted by TRIzol reagent. Total RNA (2 μg) was reverse transcribed into cDNA by RevertAid First Strand cDNA Synthesis Kit. The relative expression levels of genes were quantified using the Agilent StrataGene Mx3000P QPCR. QPCR was performed by ChamQ Universal SYBR qPCR Master Mix in a volume of 10 μl. The cycling conditions were as follows: denaturation at 95°C for 10 min, 40 cycles at 95°C for 10 s, 60°C for 15 s, and 72°C for 30 s. The cycle threshold value (ΔCq) of genes was normalized against *ACTB* and analyzed using the $2^{-\Delta \Delta Ct}$ method. Each experiment was repeated three times.

### Western Blotting

Protein was extracted from the treated cell with RIPA lysis buffer with freshly added 0.01% protease inhibitor cocktail. The Bradford method was used to calculate the content of protein. Protein from the samples were separated by 10–15% SDS polyacrylamide gel and transferred to nitrocellulose membranes. Membranes were blocked in 5% skimmed milk for 1 h. The membranes were incubated in 4°C with specific primary antibodies overnight. After washing with TBST, the blots were incubated with goat anti-rabbit secondary antibodies for 2 h at room temperature. Then the results were detected with the ECL detection reagents. Blots were normalized with a signal of *β*-actin. Each experiment was repeated three times.

### Flow Cytometric Analysis of Apoptosis

RA-FLS was treated with different concentrations of naringin, and cell apoptosis was measured using Annexin V-FITC Apoptosis Detection Kit. RA-FLS was digested and resuspended in precooled binding buffer and stained with Annexin V solution in dark for 15 min and then stained with PI solution in dark at 4°C for 15 min. Samples were subjected to flow cytometry analysis in 30 min. Each experiment was repeated three times.

### Statistical Analysis

All statistical analyses were performed using GraphPad Prism (GraphPad Software, La Jolla, CA, United States). Differences in multiple groups were analyzed by ANOVA. *p* value < 0.05 was considered statistically significant.

## Results

### Target Screening of Naringin and RA

**FIGURE 2:**
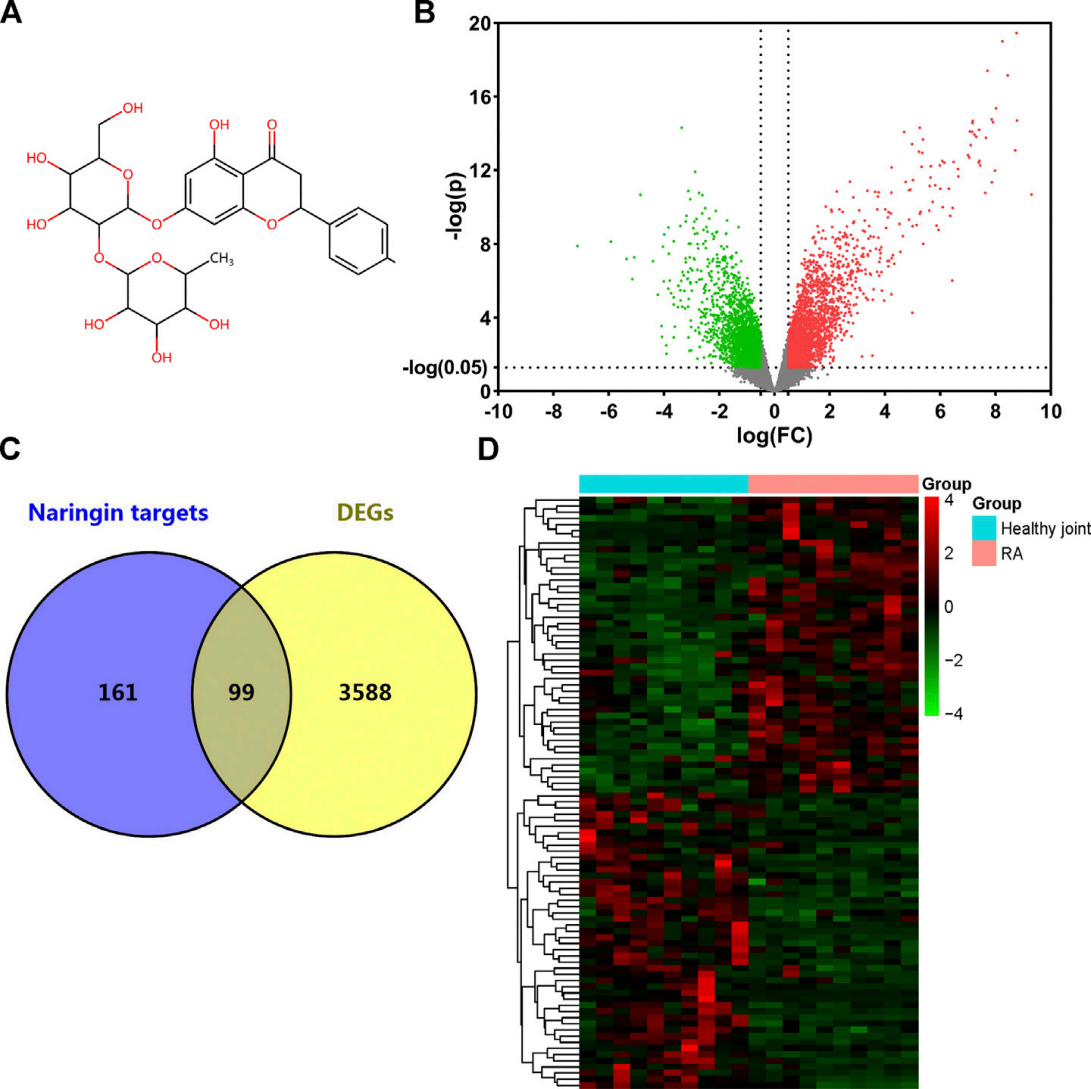
Target genes of naringin and DEGs in GSE55235. **(A)** Chemical structure of naringin; **(B)** DEGs in GSE55235 (upregulated genes were marked in red and downregulated genes were marked in green). **(C)** Venn diagram of naringin target genes and DEGs. **(D)** Clustered heat map of overlapped genes.

The 2D structure of naringin was downloaded from PubChem (Figure 2A). A total of 256 genes were identified as targets of naringin from SwissTargetPrediction database, Comparative Toxicogenomics database, and STITCH database. 3,687 DEGs were screened from GSE55235 dataset, while 1971 genes were upregulated and 1716 were downregulated (Figure 2B). Matching DEGs with naringin targets (Figure 2C), 99 genes were selected as potential targets in the therapeutic effect of naringin in RA. The heat map of these 99 genes is shown in Figure 2D.

### Enrichment Analysis of Overlapped Target

**FIGURE 3:**
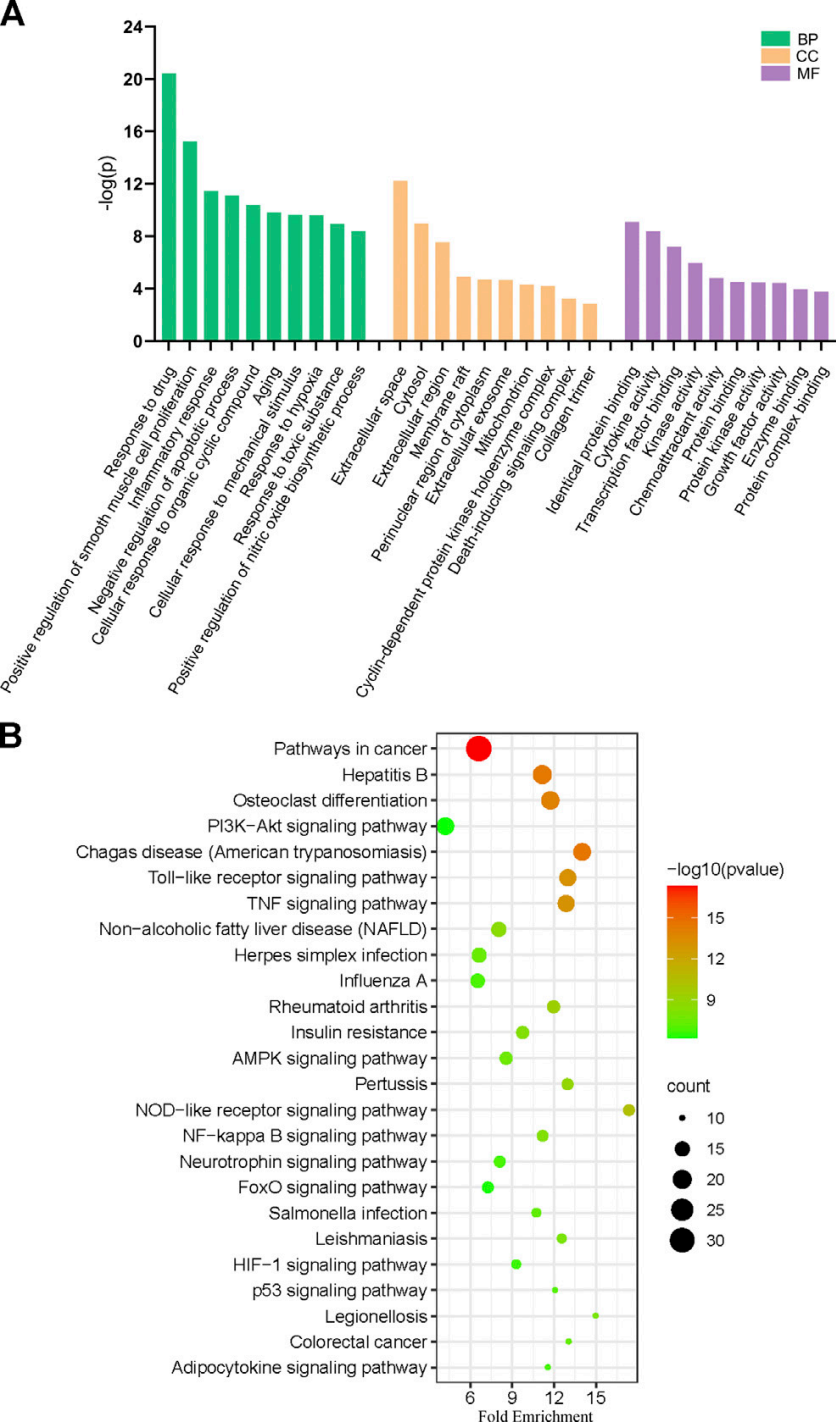
**(A)** Gene ontology (GO) enrichment analysis for key targets (top 10 were listed). **(B)** KEGG pathway enrichment analysis of key targets (top 25 were listed); the abscissa label represents Fold Enrichment of pathways.

**FIGURE 4:**
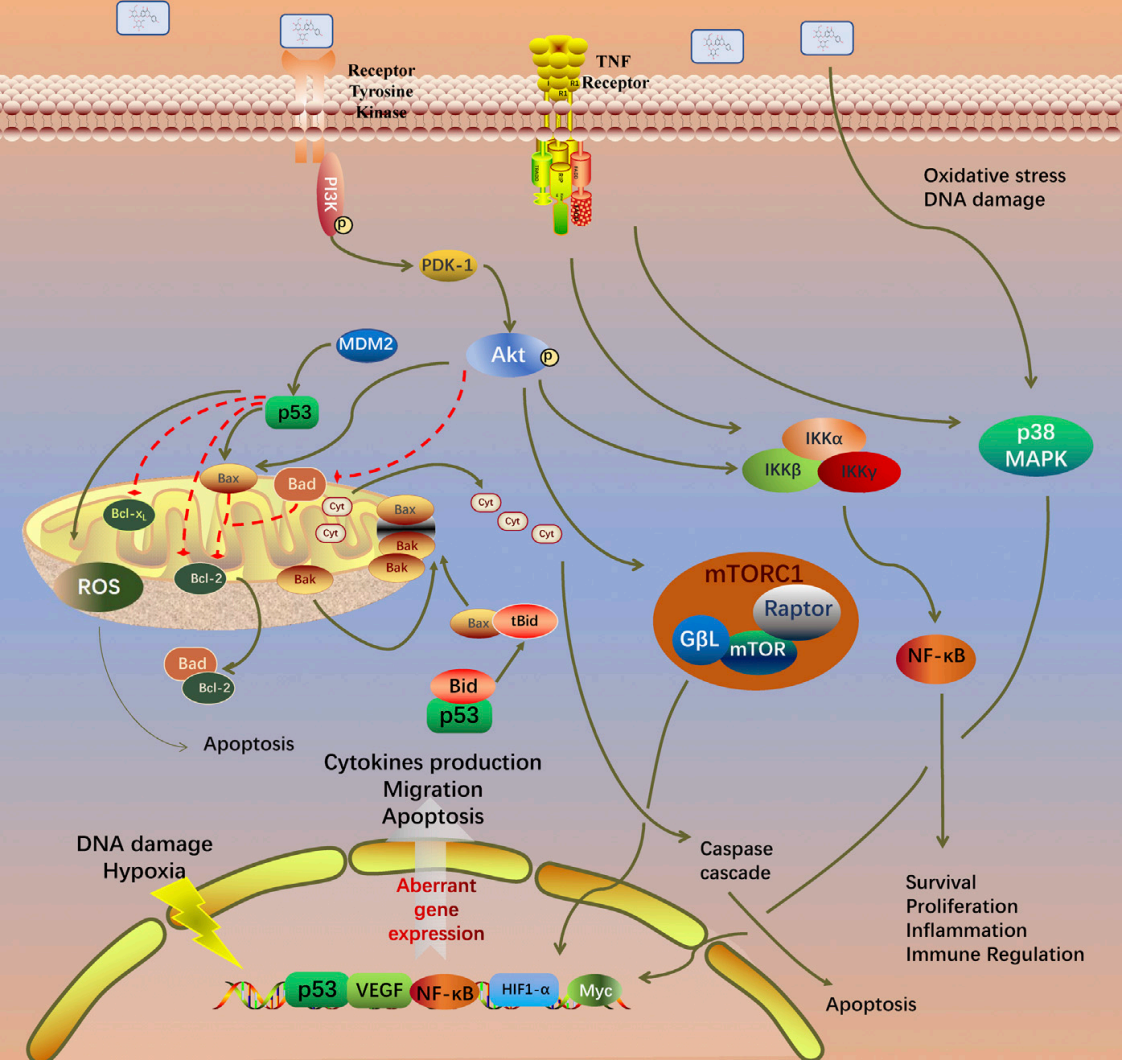
Potential naringin pathway constructed in the present research. Abbreviations: TNF-α, tumor necrosis factor alpha; TNFR, tumor necrosis factor receptor; TRADD, tumor necrosis factor receptor type 1 associated death domain protein; FADD, Fas associated *via* death domain; PI3K, phosphatidylinositol 3-kinase; PDK1, pyruvate dehydrogenase kinase 1; AKT, protein kinase B; MDM2, murine double minute2; Bcl-2, B-cell lymphoma-2; Bax, BCL2 associated X; BAD, BCL2 associated agonist of cell death; BIM, BCL2-like 11; Bak, BCL2 antagonist/killer; BID, BH3 interacting domain death agonist; MAPK1, mitogen-activated protein kinase 1; NF-κB, nuclear factor kappa B. BH3, Bcl-2 homology domain; CytoC, cytochrome C; ROS, reactive oxygen species.

GO analysis of the 99 potential therapeutic target genes was performed using the DAVID database. Target genes were mostly enriched in response to drug, positive regulation of smooth muscle cell proliferation, and inflammatory response in BP enrichment analysis; extracellular space, cytosol, and extracellular region in CC analysis; and identical protein binding, cytokine activity, and transcription factor binding in MF analysis (Figure 3A). The result of KEGG pathway enrichment analysis indicated that target genes were significantly enriched in pathways in cancer, hepatitis B, and osteoclast differentiation (Figure 3B). To further reveal the underlying mechanism of naringin in RA, a comprehensive pathway model of naringin against RA was assembled based on pathways identified through KEGG pathway analysis. Naringin may exert anti-inflammatory and proapoptotic effects by regulating p53 signaling pathway (hsa04115), apoptosis (hsa04210), HIF-1 signaling pathway (hsa04066), TNF signaling pathway (hsa04668), MAPK signaling pathway (hsa04010), NF-kappa B signaling pathway (hsa04064), and PI3K-Akt signaling pathway (hsa04151) (Figure 4).

### PPI Network Construction

**FIGURE 5:**
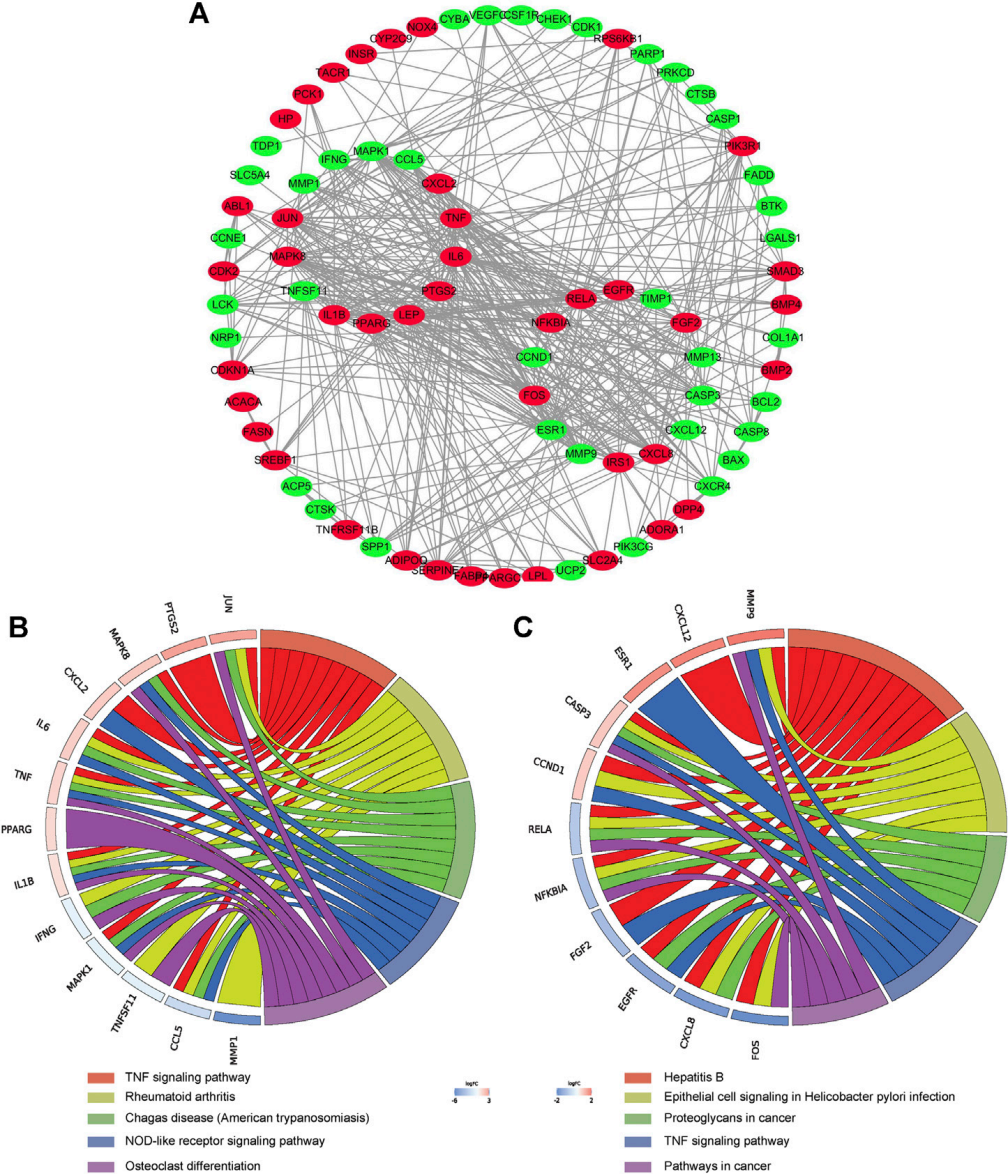
PPI network construction. **(A)** PPI network construction of key targets (subnetworks from MCODE analysis were arranged in circles). **(B) (C)** KEGG pathway analysis of genes in the subnetwork (top five were listed).

**FIGURE 6:**
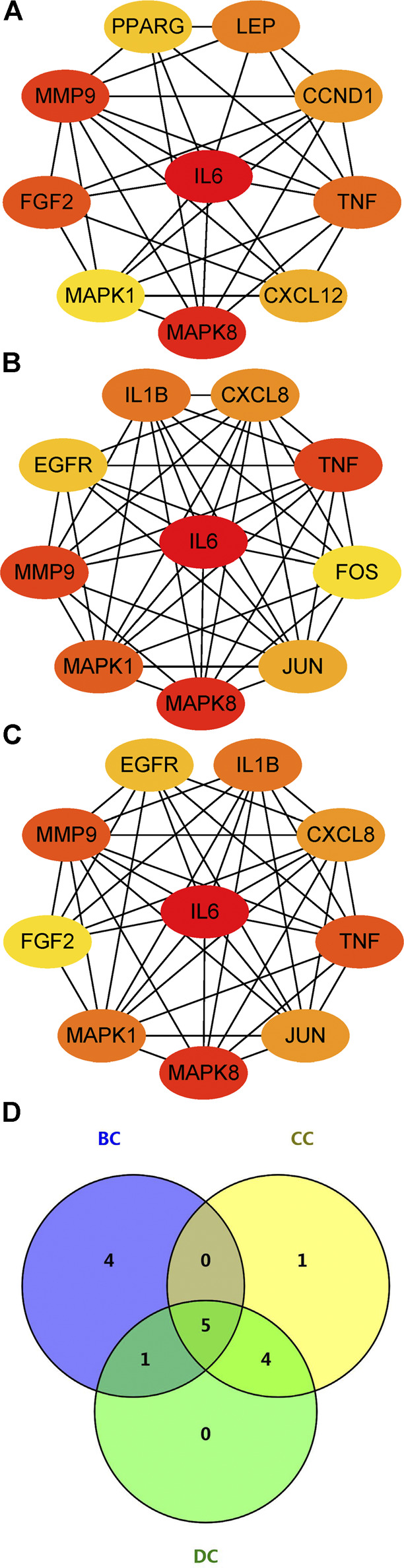
Topological analysis of key targets. **(A) (B) (C)** Top 10 genes with the highest BC, CC, and DC. **(D)** Venn diagram summarizing overlapped genes in three sections. Abbreviations: BC, betweenness centrality; CC, closeness centrality; degree centrality (DC).

The PPI network of aforementioned target proteins was constructed using STRING and visualized using Cytoscape. The PPI network consisted of 99 nodes and 484 edges, enrichment *p* value < 1.0e–16 (Figure 5A). Module analysis of the network was performed using the MCODE plug-in of Cytoscape, and two modules were selected according to aforementioned criteria. KEGG pathway analysis of these two modules were carried out using the DAVID database. Genes in module one were significantly enriched in TNF signaling pathway and rheumatoid arthritis. Genes in module two were enriched in pathways in cancer and hepatitis B (Figures 5B,C). Betweenness centrality (BC), closeness centrality (CC), and degree centrality (DC) of target proteins were calculated using topological analysis (Figures 6A–C). Top 10 hub nodes of BC, CC, and DC subnetworks were retrieved, and five overlapped genes (IL6, MAPK8, MMP-9, TNF, and MAPK1) were screened out as key targets (Figure 6D).

### Identification of RA-FLS

**FIGURE 7:**
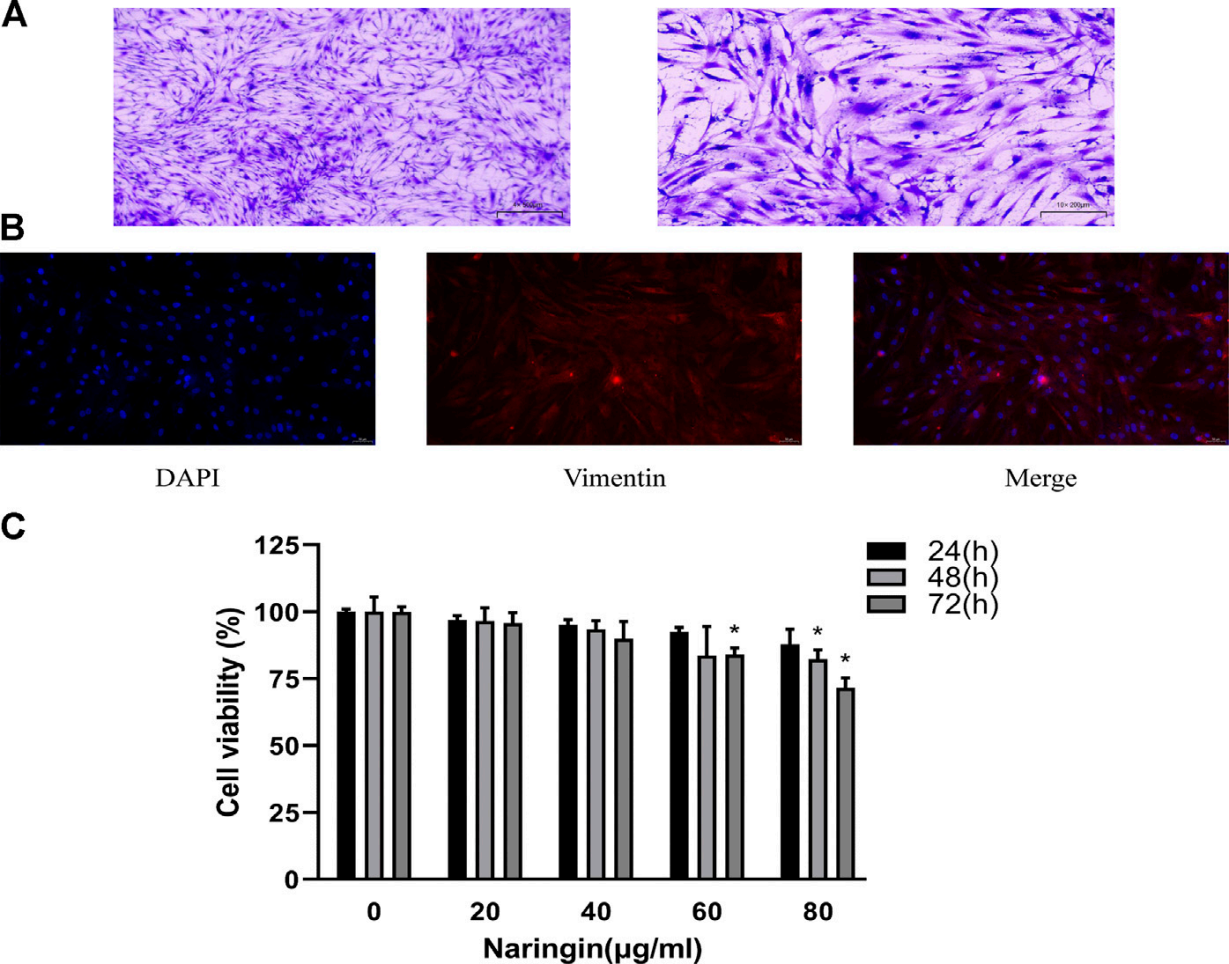
Identification of RA-FLS and therapeutic effect of naringin. **(A)** Representative images of RA-FLS stained with 5% crystal violet (100×, bar scale = 100 μm and 200×, bar scale = 50 μm). **(B)** Representative images of immunofluorescence staining for Vimentin (200×, bar scale = 50 μm). **(C)** Cell viability was measured using CCK-8 assay. Naringin reduces cell viability of RA-FLS in a dose-dependent manner. *p < 0.05. Each experiment was repeated for three times in different individuals.

Synovial tissue of RA patients was minced and digested. RA-FLS was cultured in the DMEM with 10% FBS and 1% penicillin/streptomycin. The newly extracted FLS were mixed with macrophage-like synoviocytes (MLS). At the passage three, proliferation ability of MLS disappeared, and the remaining cells were basically RA-FLS. The passage three cells were fusiform and arranged in bundles; nucleus of the cell was in the middle of cell (Figure 7A). Immunofluorescence staining showed that the cultured cells presented positive expression of vimentin (Figure 7B), which were accorded with the characteristics of FLS. The purity of passage three RA-FLS was greater than 99%.

### Naringin Decreases Cell Viability of RA-FLS

To evaluate the effect of naringin in RA-FLS, cell viability assay was carried out using CCK-8 following concentration of 0–80 μg/ml for 24–72 h. As shown in figure, treatment of RA-FLS with 80 μg/ml naringin significantly decreased cell viability (*p* < 0.05) in a time-dependent manner (Figure 7C). Thus, 80 μg/ml was the maximum dose in subsequent experiments.

### Naringin Enhances Apoptosis of RA-FLS

**FIGURE 8:**
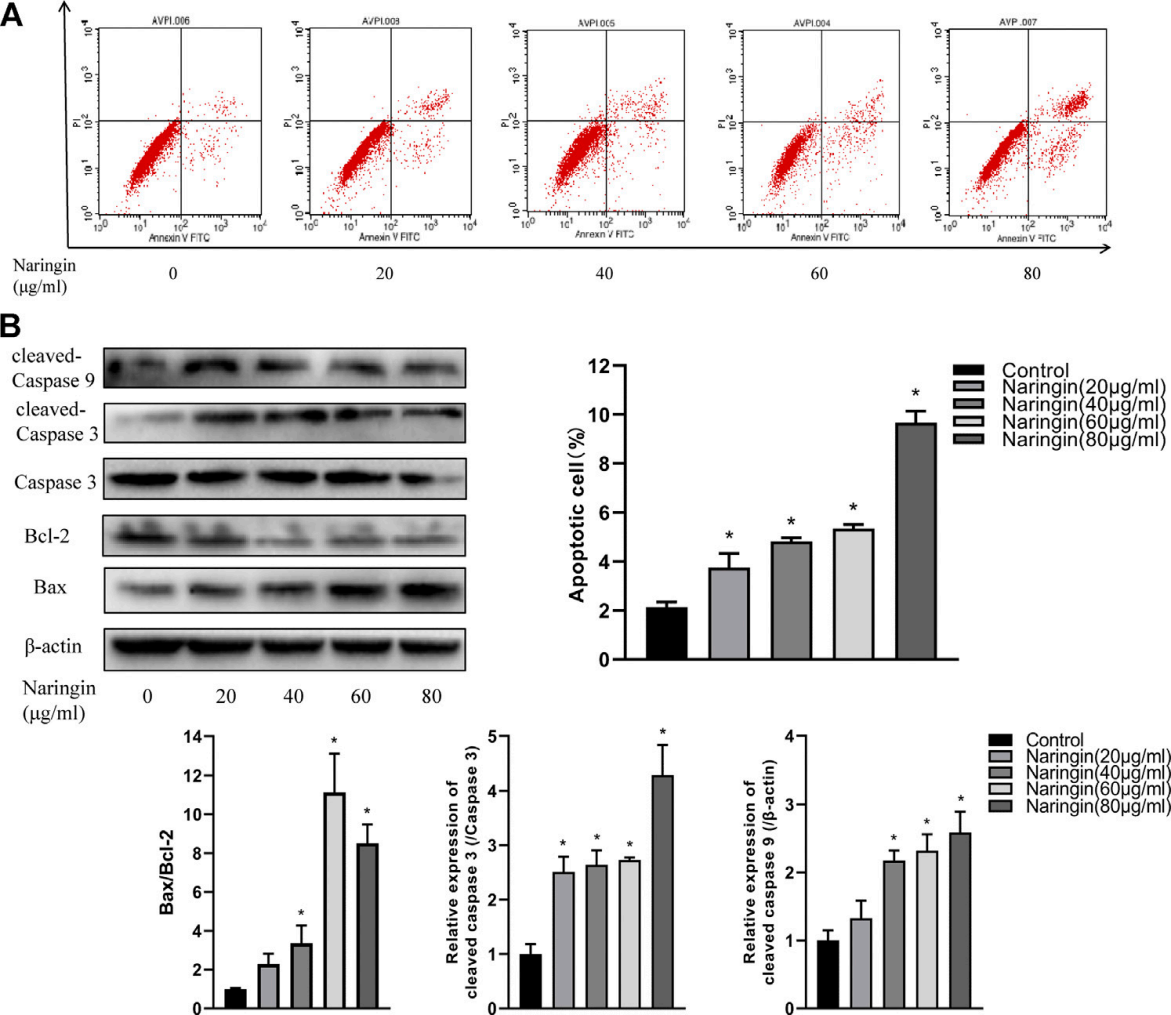
Naringin induces apoptosis of RA-FLS in a dose-dependent manner. **(A)** Representative flow cytometric plots and statistical graph. **(B)** Effect of naringin on apoptosis-related proteins expression *via* Western blot analysis and statistical graphs. *p < 0.05. Each experiment was repeated for three times in different individuals.

To quantitatively measure the effect of naringin on apoptosis in RA-FLS, RA-FLS was double stained with Annexin V-FITC/PI and measured via flow cytometry. After incubating in various concentrations of naringin, the percentage of apoptotic cells was found to be gradually increased (*p* < 0.05) (Figure 8A). Subsequently, we hypothesized whether naringin could affect apoptosis-related proteins. As expected, the expression of Bax, cleaved caspase-9, and cleaved caspase-3 were increased and anti-apoptosis protein Bcl-2 was decreased (*p* < 0.05) dose dependently (Figure 8B).

### Naringin Inhibits MMP Production and Inflammatory Cytokine Production in TNF-α–Induced RA-FLS

**FIGURE 9:**
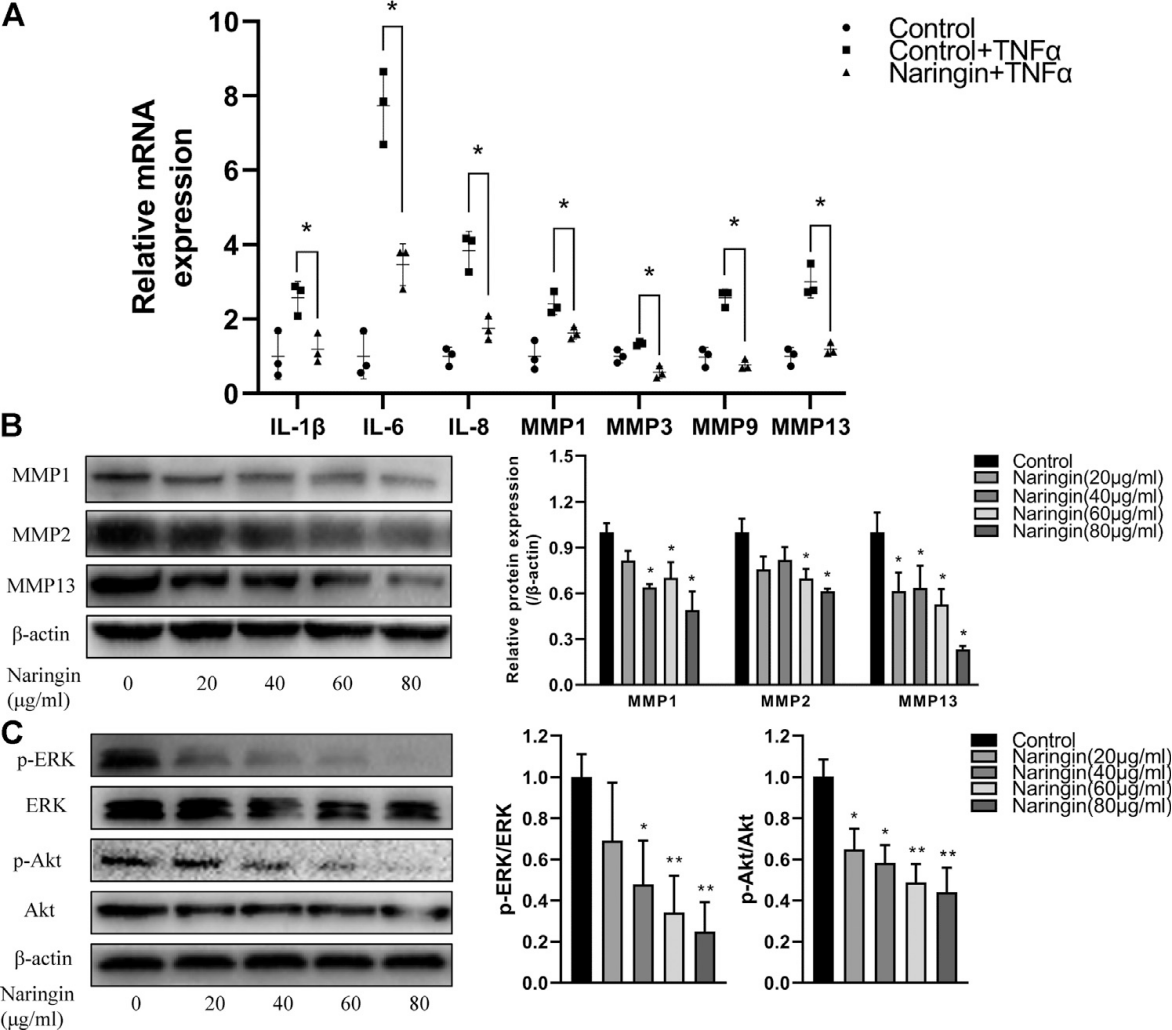
Naringin inhibits inflammatory cytokines, MMPs and *p*-Akt expression in RA-FLS. **(A)** Naringin inhibits inflammatory cytokines and MMPs expression in TNF-α induced RA-FLS. **(B)** Naringin dose dependently inhibits MMPs expression in RA-FLS. **(C)** Naringin inhibits *p*-ERK and *p*-Akt expression in RA-FLS. *p < 0.05 and **p < 0.01. Each experiment was repeated for three times in different individuals.

To investigate the anti-inflammatory effect of naringin, RA-FLS were stimulated with TNF-α after incubated with different concentrations of naringin for 24 h. We evaluated the expression of inflammatory cytokines using qPCR. After normalizing with ACTB, the expression level of IL-1β, IL-6, IL-8, MMP-1, MMP-3, and MMP-13 was significantly increased in the TNF-α group compared with the control group; the expression level of these inflammatory cytokine and MMPs was significantly reduced (*p* < 0.05) in the naringin-treated group compared with the TNF-α group (Figure 9A). The Western blot analyses indicated that naringin inhibited MMP-1, MMP-2, and MMP-13 expression (*p* < 0.05) in a dose-dependent manner (Figure 9B).

### Naringin Inhibits Phosphorylation of Akt and ERK

To further validate the network pharmacology prediction of naringin in RA-FLS, we performed Western blot to measure the phosphorylation level of Akt and ERK, and the result indicated that naringin also inhibits the expression level of *p*-Akt and *p*-ERK (*p* < 0.05) in a dose-dependent manner (Figure 9C).

## Discussion

RA is an insidious autoimmune inflammatory disease of the joints, but its etiology remains unclear (Chen et al., 2020). The treatment of RA has been transformed by the use of biologic medicines (Buch, 2018). With the combined use of traditional DMARDs and biological reagents, remission is presently an achievable goal, especially for DMARDs no-responders and newly diagnosed RA patients (Goll and Kvien, 2020). Therefore, exploring drugs against RA is very necessary. Natural products have been one of the most important major resources for pharmaceutical research and development over the past few decades, especially for the treatment of autoimmune diseases. Naringin is a disaccharide derivative (Chen et al., 2016) and has been reported as a metabolite, an antineoplastic agent, and an anti-inflammatory agent. Naringin has been reported to regulate the expression of Bcl-2 and Bax in adjuvant-induced arthritis (AIA) (Zhu et al., 2015). Oral administration of naringin to the mice with collagen-induced arthritis (CIA) alleviated joint inflammation and clinical symptoms (Kawaguchi et al., 2011). But the underlying mechanism of naringin in the treatment of RA remains unclear. Network pharmacological analysis is a relatively new approach to explore the therapeutic effect and underlying mechanism of drugs based on the network of drug and targets (Kibble et al., 2015). In the present research, we used network pharmacology to investigate the therapeutic targets involved in the naringin treatment of RA. GO, KEGG pathway analysis, and PPI network analysis of therapeutic targets were constructed. Experimental validation verified speculation on the therapeutic effect of naringin in RA.

First, a total of 99 therapeutic targets were screened out from targets of naringin and DEGs in GSE55235. Afterward, target-related enrichment analyses of GO and KEGG pathways were carried out, indicating that therapeutic efficacy of naringin on RA was a combination of multiple biological process and pathways. Among the enriched pathways, several have been reported to involve in pathogenesis of RA, as shown in Figure 4 and Supplementary Table S2.

The PI3K/Akt signaling pathway is involved in multiple pathologic changes of RA, including synovial inflammation, cartilage destruction and bone erosion, and synovial pannus formation (Malemud, 2015). Activation of PI3K/Akt signaling pathway regulates the anti-apoptotic properties and inflammatory cytokines production in RA-FLS. mTOR is the key regulator of autophagy in cancer cells. Naringin promoted apoptosis of colorectal cancer by inhibiting the PI3K/AKT/mTOR signaling pathway (Cheng et al., 2020). Naringin improves the function of HUVECs under high glucose stress through activation of the PI3K-Akt-mTOR pathway to inhibit autophagy (Wang et al., 2020). In the present study, *in vitro* experiments verified the result of KEGG pathway analysis, in which the *p*-AKT expression decreased in a dose-dependent manner.

In RA patients, increased expression of pro-inflammatory cytokines, such as TNF-ɑ, IL-1β, and IL-6, promotes osteoclastogenesis. Apraxia of joints, increased bone destruction, and insufficient done formation inevitably lead to osteoporosis and joint erosion. Osteoclast differentiation is regulated by various cytokines through the receptor activator of the NF-κB ligand complex (RANK/RANKL) and macrophage colony-stimulating factor (M-CSF) (Chambers, 2000). Naringin has been reported to exert anti-osteoporosis effects through inhibiting RANKL mediated IκB degradation and promoting OPG release from osteoblast (Liu et al., 2017). Thus, we speculated that naringin may alleviate bone destruction and promote osteogenesis in RA.

Through the PPI network, two modules were selected using MCODE analysis. Cluster-1 consisted of 14 genes. KEGG pathway enrichment analysis indicated that genes in cluster-1 enriched in TNF signaling pathway and rheumatoid arthritis. Previous research revealed that naringin suppressed the secretion of TNF-α and IL-6 induced by titanium particles in fibroblasts from the periprosthetic membrane (Yang et al., 2020b). Cluster-2 consisted of 14 genes and significantly enriched in pathways in cancer and hepatitis B. Topological analysis provides multiple complementary measurements to explore the key targets of network. Degree centrality indicates the connection “popularity” of nodes. Betweenness centrality indicates the number of shortest path passing through the nodes, while closeness centrality indicates the average distance from one node to all other nodes (Hansen et al., 2011). Comprehensive analysis of these three parameters, IL6, MAPK8, MMP-9, TNF, and MAPK1 were selected as key targets from the PPI network.

IL-6, along with IL-1 and TNF-α, belongs to cytokine family, is the key regulator of joint inflammation and damage (Hendler et al., 2010). Patients diagnosed with RA often face several comorbidities such as rheumatoid cardiovascular disease and rheumatoid lung disease, which are mostly correlated with IL-6 (Batún-Garrido et al., 2018). Continually expanding repertoire of biologic disease-modifying antirheumatic drugs (bDMARDs) allows the option of switching current treatment if it is not effective (Kearsley-Fleet et al., 2018). In our research, IL-6 shared the highest connection degree among all targets. The qPCR result revealed that naringin inhibited the mRNA expression of IL-6, and IL-8 in TNA-ɑ induced RA-FLS. Thus, we speculate that naringin may act as a natural anti-inflammatory agent in RA.

Several studies revealed that the MAPK/ERK signaling pathway regulates cell differentiation and apoptosis (Mcarthur, 2015). MAPK8, also known as Jun nuclear kinase (JNK), was reported to bind c-Jun and enhance its transcription in response to environmental stress, radiation, and various growth factors (Johnson, 2002). KEGG pathway analysis indicated that target genes were significantly enriched in the MAPK signaling pathway. Previous research revealed that naringin mitigated cardiac hypertrophy by inactivation of JNK(Adebiyi et al., 2016). MAPK1, also known as ERK, regulates Bcl-2 expression and activates caspase cascade (Zhang et al., 2019). Abnormal activation of MAPKs in synovial tissues of RA patients promotes pannus formation (Ding, 2006). Consequently, MAPK inhibitors are considered as promising potential agents in treatment of RA. The Western blot result indicates that naringin inhibits expression of *p*-ERK dose dependently. Naringin exerts anti-RA effect as a natural ERK inhibitor.

Matrix metalloproteinases (MMPs) are metalloendopeptidases with diverse structure domains (Itoh, 2017). Infiltration of inflammatory cell in the joint cavity leads to overwhelming release of pro-inflammatory cytokines such as IL-1β and TNF-α. Cytokines stimulate the production of MMPs such as MMP-1, MMP-3, MMP-9, and MMP-13 (Burrage et al., 2006). This vicious cycle eventually leads to irreversible damage to articular cartilage and bone. MMP-9 expression was mediated by activation of MAPK and PI3K/Akt signaling pathways (Cho et al., 2000; Jin et al., 2008). In our research, MMP-9 was selected as a key target in the treatment of RA. It has been reported that naringin inhibits the expression of MMP-2 and MMP-9 in human glioblastoma (Aroui et al., 2016). Naringin alleviates articular cartilage and bone erosion in RA by inhibiting synovial tissue secretion of MMPs. Experimental results also indicated the inhibition of naringin in MMP production.

However, *in vivo* experiments are necessary to evaluate the therapeutic effect of drugs. It has been reported that oral administration of naringin to the mice with CIA alleviates knee inflammation and clinical symptom severity (Kawaguchi et al., 2011). The metabolic process of naringin after oral administration needs to be studied. Naringin was hydrolyzed to naringenin in the digestive tract before absorption (Ameer et al., 1996), and naringenin suppressed the immune response and disease activity through impairing dendritic cell maturation *in vitro* and *in vivo* (Li et al., 2015).

## Conclusion

In this study, therapeutic effects and underlying mechanisms of naringin against RA were elucidated *via* network pharmacology and *in vitro* experimental validation. We demonstrated that naringin inhibits inflammatory cytokines production and promotes apoptosis in RA-FLS *via* PI3K/Akt and MAPK/ERK signaling pathways. Our research also suggested that network pharmacology combined with experimental validation provides a novel approach to investigate the potential targets of traditional Chinese medicine.

## Data Availability

The datasets presented in this study can be found in online repositories. The names of the repository/repositories and accession numbers can be found in the article/Supplementary Material.
